# Supplementary material for: Analysis of factors influencing levee safety using the DEMATEL method
Source: PLoS One. 2021 Sep 10;16(9):e0255755. doi: 10.1371/journal.pone.0255755 (PMC8432840; doi:10.1371/journal.pone.0255755)
Supplement: Attachment — Submitted filename: Response_to_reviewers.docx [file pone.0255755.s002.docx]

July 8^th^, 2021

Warsaw University of Life Sciences

Department of Hydrology,

Meteorology and Water Management

Nowoursynowska 166, 02-787 Warsaw, Poland

Dorota Mirosław-Światek, PhD

Dear Dr Ahmed Mancy Mosa

Academic Editor,

We submit the revised manuscript entitled “*Analysis of factors influencing levee safety using the DEMATEL method”* to be considered for publication in PLOS ONE.

We would like to thank the Editor and the anonymous Reviewers for their valuable and constructive comments that have allowed to improve this paper. All comments have been carefully considered and the manuscript has been revised and corrected accordingly. All changes are included in this accompanying letter and are also in the revised manuscript.

We continue to believe that the manuscript would be of interest to the diverse readership of PLOS ONE.

Thank you for your consideration.

Yours sincerely,

Dorota Mirosław-Świątek

(on behalf of the authors)

**Reviewer 1**

We would like to thank the Reviewer for the work done in this revision. We will follow and answer all the proposed questions/issues to correctly improve the manuscript.

They use a methodology, which is not novel, and apply it to study the safety level of levees. The safety is assessed by means of experts’ knowledge. Therefore, the authors do not quantify this safety level. This is the main drawback of the paper. In order to prove the results obtained in the study, they should be related to some other studies performed on the assessment of levees conditions. The manuscript could improve its scientific base and novelty by including information regarding other studies but also, by including the results of those studies in the elaboration of matrices, if possible.

We would like to note kindly that the aim of our study was to find out the inter-relationships of factors impacting the levee technical condition performed as a cause-and-effect model and not to develop a method for assessing levee safety of levees conditions. The obtained results from our the causal-and-effect model allowing through the application of the DEMATEL method, the identifying cause-and-effect relationships between those factors and classifying them as cause or effect factors. The results of this type of analysis have not been presented in the literature so far. This approach cannot be interpreted as a method for evaluating the performance of levees. Therefore, our results cannot be related to some other studies performed on the assessment of levees conditions. In revised manuscript the Discussion has been rewritten as suggested by the Reviewer. We clarified better the context of our analyses in relation to levee performance evaluation methods. The following text (lines 374-424) was added.

*Levee failure can result from different type of break mechanisms: internal erosion, overflow, downstream slide, upstream slide and scouring [35]. Each mechanism consists in a sequence of component failures leading to the levee break and is related to the state of eight factors whose model of cause-and-effect relationships we have developed. Generally levee safety assessment is a process at which we evaluate the probability of levee failure, and methods for assessing levee safety are widely discussed in the literature. A wide range of methods are used in assessing levee safety, from the relatively simple based on expert assessment, through index-based methods and empirical models for levee failure modes, to the very complex physical and mathematical models of levee failure modes [36]. A common approach is where the engineer/expert evaluates qualitatively the performance of levees based on heuristics (knowledge, intuition and experience) [37]. In many cases, experts make levee safety evaluation through probabilistic risk assessment of failure of these structures through direct expression of the numeric data. Fauchard [38] described the principles of a method developed on levees in France that can be successfully applied to levees in other countries around the world. The method usually starts by collecting as much information as possible about the levee based on archival analyses, interviews with managers and a site visit. This phase is essential to ensure the high quality of the final diagnosis. The second stage - geophysical surveys - aims to identify areas of the levee which, due to their specific physical characteristics, are vulnerable to damage during floods. The geophysical methods used must be able to survey over long distances and reveal inhomogeneity both within the levee and its foundation. The third phase, geotechnical investigations - soundings and borings - allow the mechanical characteristics and properties of the materials used to construct the levee to be determined. This approach is representative of those responsible for the safety of flood embankments, in particular, for owners, managers, engineers and contractors. With this in mind, Tourment [39] proposed an analysis of structural failure modes that can help to identify different scenarios of levee failure - and properly evaluate the safety status. The proposed three different assessment methods are based on combining data, expert judgments, index based methods and empirical (physical and mathematical) models. Peyras [40], in contrast, develop methods that can assist levee safety using geographic information system (GIS). Levee safety is closely linked to levee technical condition, which is evaluated using numerous publications in the form of handbooks or guidelines (e.g. [7], [10], [35]), with the most famous being The International Levee Handbook (ILH) [2]. The assessment is based on expert knowledge supported by the results of measurements, analyses, calculations and modelling. The guidelines, depending on the significance class of a hydrotechnical structure, thoroughly define the test, measurement and evaluation procedure, which covers surveying, geotechnical measurement, laboratory analyses and mathematical modelling. The guidelines also contain tips regarding the types and scopes of modelling calculations, starting with calculating slope stability coefficients, and ending with seepage and suffosion potential calculations. Based on the conducted analysis, a traditional levee technical condition evaluation involves the qualitative or quantitative determination of parameters, which characterize the factors (F1-F8) we analysed. Although their inter-relationships are analysed in the performance of levees in the form of sequences leading to levee failure, they are not take into account in the form of the causal-and-effect model that we have developed for these factors. This model cannot, therefore, be regarded as a method to assess* *levee safety or technical condition. The results we have obtained, allowing through the application of the DEMATEL method, the identifying cause-and-effect relationships between those factors, depend on the form of the direct-influence matrix A, that has been formulate by experts, using an integer scale (0 - 3). A qualitative assessment based on the knowledge, intuition and experience of experts is a common approach in the performance of levees. For example, in the levee safety assessment method developed by Vuillet [37], expert/engineer evaluates analysed criterion of levee performance according to the evaluation scale of 0 to 10. Expert evaluation is also widely used in applications of the DEMATEL method. In an article by Si [17], in which authors reviewed a total of 346 papers on the application of DEMATEL published from 2006 to 2016 in the international journals, only in one case was the direct-influence matrix A, developed not on the basis of expert opinion, but as a result of applying the relations map (RM) obtained from the artificial neural network (ANN) [41].*

In the revised manuscript the following articles have been added to the references:

1. Mériaux P, Royet P, Folton C. 2001. Surveillance, entretien et diagnostic des digues de protection contre les inondations [Monitoring, maintenance and diagnosis of dikes: A practical guide for owners and managers]. Paris: Cemagref ed.
2. FloodProbe. 2012. CombiningInformationforUrbanLevee Assessment, ReportNumber:WP03-01-12-24.Availablefrom: <http://www.floodprobe.eu>
3. Vuillet M, Peyras L, Carvajal C, Serre D, Diab Y. 2013. Levee performance evaluation based on subjective probabilities, European Journal of Environmental and Civil Engineering, 17:5, 329-349, DOI: 10.1080/19648189.2013.785723
4. Fauchard C, Mériaux P. 2007. Geophysical and Geotechnical Methods for Diagnosing Flood Protection Dikes, Guide for implementation and interpretation.
5. Tourment R., Beullac B., Sab G A. 2019. Levee safety assessments using an index-based method. XVII ECSMGE-2019, Geotechnical Engineering foundation of the future, Sep 2019, Reykjavik, Iceland. pp.8.
6. Peyras L, Tourment R, Vuillet M, Beullac B, Delaunay C., Bambara G. 2017. Development of an expert-led GIS-based approach for assessing the performance of river levees: the D igsure method and tool. Journal of Flood Risk Management 10:3, pages 393-407.
7. Chen Y, Liu J, Li Y, Sadiq R, Deng Y. 2015.RM-DEMATEL: a new methodology to identify the key factors in P M 2.5, Environmental Science and Pollution Research, vol. 22, no. 8, pp. 6372–6380.

There is no mention about the piping effect, which is important in the safety assessment of levees.
Hydraulic piping is one of the most most dangerous phenomenon, which leads to a levee failure. It was not included as a factor influencing levee technical condition because its mechanism of occurrence is related to a sequence of events resulting from the condition of factors F7 - condition of levee top and slopes (maintenance work, filtration deformations, settlement) and F8 - hydrological factor, which were included in our analysis. Its mechanism of formation in relation to the F7 and F8 factor is explained in revised manuscript. The following text (lines 227-238) was added to the Basic factors influencing levee technical condition in the section describing the characteristics of the F7 factor.

*The conditions of water seepage through the levee body and subsoil, and the associated phenomena, greatly influence its safe operation. In the event of maximum river water flow rates and levels, usually, within the first few days, water passage into the levee structure is based on filtration. After this period, if the structure still experiences high water levels, seepage occurs primarily through the subsoil, and also commences through the levee body. Water movement in the subsoil ground and levee body can cause the formation of water exudation and effusion zones onto the landside slope and the landside, leading to harmful seepage deformation phenomena [32]. A cross-case analysis based on the course of physical phenomena leading to seepage deformations, as well as their descriptions and definitions was thoroughly discussed in the work [33]. Initial seepage deformations, such as erosion or suffosion can lead to the formation of the most dangerous phenomenon, namely, hydraulic piping. This is why observing the initiation of seepage deformation phenomena is crucial in terms of evaluating levee condition.*

**Reviewer 2**

We would like to thank the Reviewer for the work done in this revision. We will follow and answer all the proposed questions/issues to correctly improve the manuscript.

The authors did a good work and covered an interesting area. However, only few remarks can be addressed to improve the quality of the article: Conclusions: Rewrite the conclusions in Points. Double check the references and unify the citation style in the list

As suggested by the Reviewer we have rewritten the Conclusions in points. In the revised manuscript we have corrected the citation style the reference.
